# Supplementary material for: Nonadiabatic Surface Hopping Dynamics of Photocatalytic Water Splitting Process with Heptazine–(H2O)4 Chromophore
Source: Int J Mol Sci. 2025 May 9;26(10):4549. doi: 10.3390/ijms26104549 (PMC12111505; doi:10.3390/ijms26104549)
Supplement: Supplementary file 1 [file ijms-26-04549-s001.zip › ijms-3595756-supplementary.pdf]

Supporting Information

Nonadiabatic Surface-Hopping Dynamics of Photocatalytic Water Splitting Process with Heptazine-$(H_2O)_4$ Chromophore

Xiaojuan Pang, Chenghao Yang,Ningbo Zhang and Chenwei Jiang

Table S1. The Cartesian coordinates of ground-state equilibrium geometries of heptazine -$(H_2O)_4$ clusters calculated with MP2 method with cc-pVDZ mixed with aug-cc-pVDZ basis set.

C	-0.0436112	-0.2458069	1.6529839
C	-0.3172479	1.9731116	2.1387364
C	-2.1905093	0.8800690	1.4579575
C	-3.9637149	-0.3585370	0.7637771
C	-1.9997006	-1.4896529	0.9172110
C	0.0405705	-2.4702501	1.1244625
H	0.6592926	-3.3711433	1.0394811
H	0.1539831	2.9053518	2.4711601
H	-5.0322884	-0.3893281	0.5216378
N	-1.4103788	-0.2850980	1.3429085
N	-1.6213023	2.0309665	1.8653619
N	-3.4958404	0.8335245	1.1600101
N	-3.3063352	-1.5182067	0.6226987
N	-1.2485535	-2.6026031	0.8094106
N	0.6891928	-1.3644010	1.5371783
N	0.5053135	0.9096090	2.0596143
O	3.5452700	-1.8521441	2.0075602
H	3.7022499	-1.6109132	2.9438126
H	2.5993971	-1.6433527	1.8869220
O	3.2483757	1.5443014	2.8902448
H	2.3592223	1.2890918	2.5787110

H	3.8183313	1.3211743	2.1254440
O	3.6762078	-0.6094081	4.5235950
H	3.5917153	0.2578045	4.0582272
H	4.3642151	-0.4796121	5.1868434
O	4.5527351	0.3391906	0.7135388
H	4.3117660	-0.5363715	1.1021267
H	5.4732601	0.2542094	0.4387155
